# Natural Killer Cell Count in Systemic Lupus Erythematosus Patients: A Flow Cytometry-Based Study

**DOI:** 10.7759/cureus.46885

**Published:** 2023-10-12

**Authors:** Nirvana Thangjam, Biswajit Dey, Yookarin Khonglah, Iadarilang Tiewsoh, Rosina Ksoo

**Affiliations:** 1 Pathology, North Eastern Indira Gandhi Regional Institute of Health and Medical Sciences (NEIGRIHMS), Shillong, IND; 2 Pathology and Laboratory Medicine, North Eastern Indira Gandhi Regional Institute of Health and Medical Sciences (NEIGRIHMS), Shillong, IND; 3 Internal Medicine, North Eastern Indira Gandhi Regional Institute of Health and Medical Sciences (NEIGRIHMS), Shillong, IND; 4 Pediatrics, North Eastern Indira Gandhi Regional Institute of Health and Medical Sciences (NEIGRIHMS), Shillong, IND

**Keywords:** cd16, cd56, disease activity, systemic lupus erythematosus, natural killer cells

## Abstract

Background

Natural killer cells (NK cells) are important mediators of innate immune regulation and literature has shown that they have a role in shaping the adaptive immune system.

Objective

The present study was undertaken to analyze the NK cell count in systemic lupus erythematosus (SLE) patients as compared to that of controls.

Materials and methods

Safety of Estrogens in Lupus Erythematosus National Assessment SLE Disease Activity Index (SELENA-SLEDAI) score was assessed in 32 SLE cases. CD3(-) cells were identified as NK cells on flow cytometry, and then their subsets CD56(+) and CD16(+) cells were identified compared to 30 healthy controls. Receiver Operating Characteristic (ROC) curve analysis was performed on NK cells to attempt to determine a cut-off point.

Results

The CD3(-) NK cells, including the percentages of CD56(+) and CD16(+), were significantly (p<0.001) reduced in SLE patients (12.35%, and 18.7%) as compared to controls (24.67%, and 46.6%). On ROC curve analysis, cut-off values <481/cumm with sensitivity of 86.7% and specificity of 84.4% for CD3(-) NK cells (p<0.001), <23% with 60% sensitivity and 75% specificity for CD56(+) NK cells (p<0.001), and <29% with sensitivity of 70% and specificity of 87.5% for CD16(+) NK cells (p<0.001) were noted. Subsets of NK cells showed no association with the clinicopathological parameters like age, sex, disease activity, anti-nuclear antibodies (ANA), dsDNA, absolute lymphocyte count, and renal involvement.

Conclusion

NK cells, and their subpopulations of CD56(+) and CD16(+) cells, are decreased in patients with SLE as compared to controls.

## Introduction

Systemic Lupus Erythematosus (SLE) is the prototypic systemic autoimmune disease, characterized by a lack of tolerance to nuclear autoantigens, enhancement of clones of autoreactive T and B cells, and activation of the polyclonal B cell. All these lead to hypergammaglobulinemia, increased autoantibody production, deposition of immune complexes, and inflammation of the systemic tissues [1]. The onset of the disease can be abrupt or insidious, and it is usually a chronic, remitting, and relapsing illness that is generally febrile. Almost every organ in the body can be harmed.

Studies have verified that the pathogenic production of autoantibodies against autoantigens by B cells is promoted with the help of T cells, especially cluster of differentiation (CD4)(+) T cells. In addition to B and T cells, natural killer (NK) cells of the innate immune system have also been reported to play a role in the development of pathogenic autoantibodies in lupus by helping B cells [2].

NK cells are an important link between the innate and adaptive immune systems; variations in their activity correlate with several autoimmune diseases [1]. Reports from several groups show significantly lower NK cell numbers in the blood of SLE patients compared with controls, especially in lupus nephritis patients. This has been linked to elevated serum levels of Interferon alfa (IFN-α), a cytokine that promotes activation-induced apoptosis in SLE patients [3]. Furthermore, Cho et al., 2011 found that reductions in absolute NK T cell numbers are correlated with SLEDAI (SLE Disease Activity Index), which suggests that NK T cells participate in disease activity control [4].

Keeping these in the background, we undertook the present study to compare the NK cell count of SLE patients, against age and sex-matched healthy individuals; and to establish cut-off values of different subsets of NK cells in SLE patients.

## Materials and methods

This is a hospital-based prospective analytical study, carried out for one year from May 2020 to April 2021. The study was approved by the Institute Ethics Committee at North Eastern Indira Gandhi Regional Institute of Health and Medical Sciences, Shillong, India (approval number NEIGR/IEC/M10/T14/2020). A total of 32 cases of SLE who fulfilled ACR/EULAR (American College of Rheumatology/ European League Against Rheumatism) criteria were included in the study. Patients with known concurrent infectious diseases, malignancies, or acute inflammation not attributed to lupus flare were excluded from the study. All the cases were assessed for disease activity as per the Safety of Estrogens in Lupus Erythematosus National Assessment SLE Disease Activity Index (SELENA-SLEDAI). Thirty healthy controls were included in the study. Peripheral blood was collected in ethylene diamine tetraacetic acid (EDTA) vials from both the study and control groups and was subjected to flow cytometric analysis to estimate the NK cell count and identify its subsets using BD Biosciences FACS Calibur. The absolute lymphocyte count (ALC) of the cases and controls was calculated from an automated hematology analyzer (Sysmex XN 1000i; Sysmex, USA).

**Figure 1:**
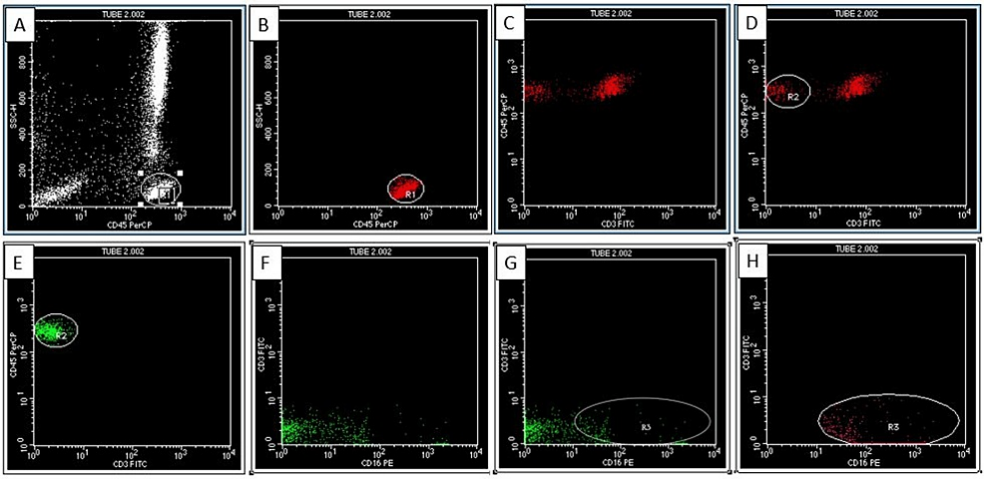
Scatter plots The lymphocyte cluster was gated on CD45 versus a side scatter plot (A, B). The cells were subsequently gated on CD45 versus CD3 and the percentage of CD3 negative cells was noted (C, D). CD3 negative NK cells were categorized based on CD16 positivity or negativity (E, F, G, H). CD: Cluster of differentiation

Flow cytometric analysis of the samples was done using CD45, CD3, CD16, and CD56 antibodies tagged with fluorochromes. A sequential gating strategy was used to analyze the NK cell subsets. The lymphocyte cluster was gated on CD45 versus a side scatter plot (Figure 1). The cells were subsequently gated on CD45 versus CD3 and the percentage of CD3(-) cells were noted. CD3(-) NK cell count was then calculated based on the percentage of CD3(-) cells and ALC. CD3(-) NK cells were categorized based on CD56(+)/(-) and CD16(+)/(-). The CD56(+) and CD16(+) cells were further classified into bright and dim based on the fluorescence intensity.

We classified the NK cells according to the maturation pattern into six subclasses in increasing order of maturity [5]: (i) CD56bright CD16(-) (most immature); (ii) CD56bright CD16dim; (iii) CD56dim CD16(-); (iv) CD56dim CD16dim; (v) CD56dim CD16bright; (vi) CD56(-) CD16bright (most mature).

The results were compared between the cases and controls. The patients were divided based on the SELENA-SLEDAI (Safety of Estrogens in Lupus Erythematosus National Assessment SLE Disease Activity Index) as mild/moderate and severe [6].

Statistical package for social sciences (SPSS), IBM SPSS Statistics 23.0 (IBM Corp., Armonk, NY) was used for statistical analysis. Mean, median, percentage, 95% confidence interval, Fisher's exact test, and Receiver Operating Characteristic (ROC) curve analysis were used. A ROC curve was plotted to attempt to determine the value of NK cell count in SLE patients compared to the control group. A p-value less than 0.05 was considered statistically significant.

## Results

Of the 32 cases, 29 (91%) cases were females while three (9%) cases were males. The female: male ratio was 9.7: 1. The patients' ages varied from 11 years to 37 years with a mean of 25 years. Table 1 indicates the frequency of clinical manifestations in SLE.

**Table 1:** Clinical manifestations of the SLE patients SLE: Systemic lupus erythematosus

Signs and symptoms	No. of cases (n=32)	Percentage (%)
Mucocutaneous	19	59.4
Constitutional	18	56.25
Renal	18	56.25
Hematological	17	53.12
Musculoskeletal	8	25
Serositis	5	15.6
Gastrointestinal	4	12.5
Pleuropulmonary	2	6.25
Neuropsychiatric	1	3.12

All the cases were positive for anti-nuclear antibodies (ANA). Twenty-five cases (78%) were positive for anti-dsDNA antibodies. Out of 18 cases with renal manifestations, 14 cases (77.8%) had a renal biopsy done, which confirmed lupus nephritis. Out of these, most of the cases (8/14, 25%) were diagnosed with class IV lupus nephritis as per the International Society of Nephrology/Renal Pathology Society classification.

There was a significant reduction (p< 0.001) in the absolute lymphocyte count (ALC) in cases as compared to controls. We also found a significant reduction (p< 0.001) in the absolute CD3(-) NK cells, percentages of CD56(+) and CD16(+) NK cells in cases compared to controls (Table 2). There was no significant association of absolute lymphocyte count (ALC), absolute CD3(-) NK cell count, CD56, and CD16 percentages with disease activity.

**Table 2:** Median values of ALC, CD3 negative cells, percentage of CD56 positive cells and percentage of CD16 positive cells (between cases and controls) ALC: Absolute lymphocyte count; CD: Cluster of differentiation

Parameters	Median value		p-value
	Cases (Range)	Controls (Range)	
ALC (Per cumm)	945 (70-5683)	5722 (2426-9663)	<0.001
CD3(-) (Per cumm)	232.5 (10-1462)	734 (341-2184)	<0.001
CD56(+) (%)	12.35 (0-93.6)	24.67 (4.8-94.2)	<0.001
CD16(+) (%)	18.7 (0-89)	46.6 (11.3-81.57)	<0.001

**Figure 2:**
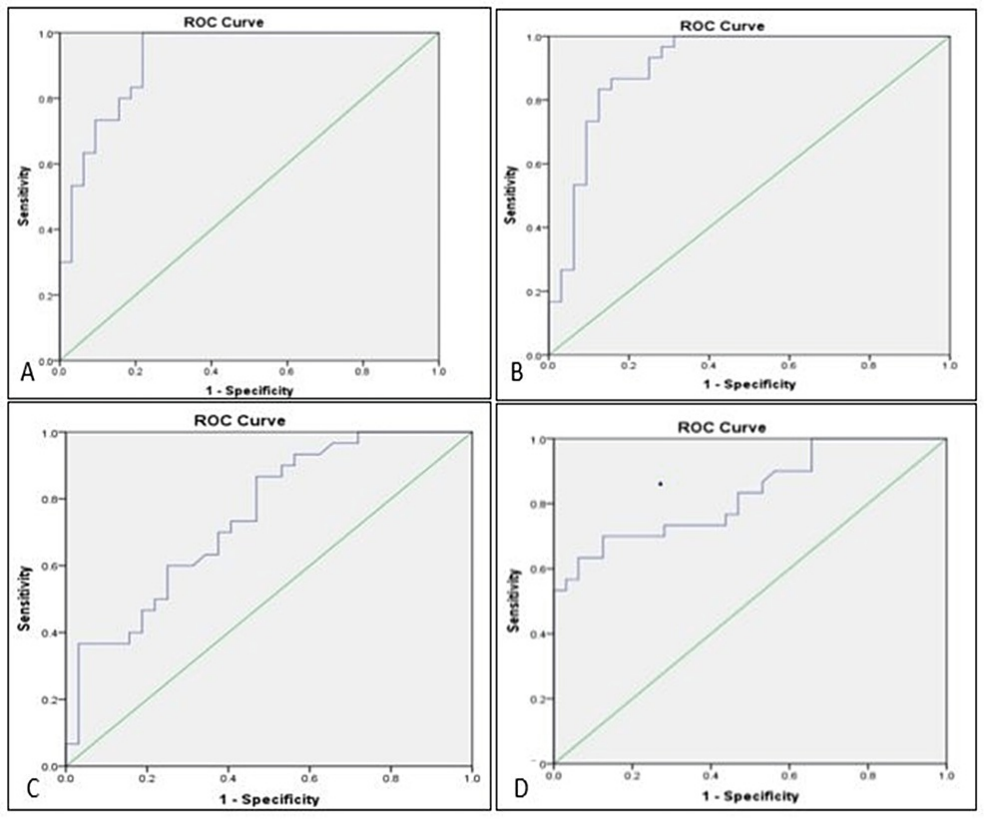
ROC curves (A) ROC curve for ALC of SLE patients compared to control (B) ROC curve for CD3- cells of SLE patients as compared to controls (C) ROC curve for % CD56+ cells of SLE patients as compared to controls (D) ROC curve for %CD16+cells of SLE patients as compared to controls ROC: Reciever operating curve; ALC: Absolute lymphocyte count; SLE: Systemic lupus erythematosus; CD: Cluster of differentiation

ROC curve analysis showed the cut-off values, the area under the curve, sensitivity, and specificity of ALC, CD3(-) cells, percentage of CD56(+) cells, and percentage of CD16(+) cells in cases compared to controls (Table 3 and Figure 2).

**Table 3:** Cut-off values, area under curve, sensitivity, and specificity of ALC, CD3 negative cells, percentage of CD56 positive cells and percentage of CD16 positive cells (between cases and controls) ALC: Absolute lymphocyte count; AUC: Area under the curve; CD: Cluster of differentiation

Variable	AUC	Cut-off	Sensitivity (%)	Specificity (%)	p-value
ALC	0.924	<2528 per cumm	96.7	78.1	<0.001
CD3(-)	0.907	<481 per cumm	86.7	84.4	<0.001
CD56(+)	0.747	<23%	60	75	<0.001
CD16(+)	0.830	<29%	70	87.5	<0.001

Of the 32 cases, 20 presented with mild/moderate flare (62.5%), eight with severe flare (25%), and four with no flare (12.5%). We observed that age, gender, ANA, anti-ds-DNA antibody, ALC, absolute NK cell count, and CD56 and CD16 percentages could not predict disease activity.

As compared to controls, SLE patients showed a higher frequency of mature CD56dim (53.4% vs 78.13%) and a lower frequency of immature CD56bright NK cells (40% vs 18.75%) (Table 4).

**Table 4:** CD56 and CD16 expression among cases and controls CD: Cluster of differentiation

Expression	CD56 (%)		CD16 (%)	
	Cases	Controls	Cases	Controls
Bright	6 (18.75%)	12 (40%)	8 (25%)	7 (23.4%)
Dim	25 (78.13%)	16 (53.4%)	17 (53.13%)	17(56.6%)
Negative	1 (3.12%)	2 (6.6%)	7 (21.87%)	6 (20%)

The cases are distributed based on the maturation pattern of NK cells and are compared to various parameters such as age, gender, ANA, anti-ds-DNA antibody, ALC, absolute NK cell count, CD56 and CD16 percentages, and renal involvement. However, there was no significant difference between CD56bright or CD56dim NK cells with the mentioned parameters.

## Discussion

SLE is a progressive autoimmune disease that can affect virtually any organ of the body. It is characterized by the presence of antinuclear antibodies. The goal of this study is to look into the number of NK cells in the peripheral blood of SLE patients as compared to controls, as well as their CD56 and CD16 expression. The clinicopathological profile of SLE patients is also analyzed with respect to the levels of these cells.

SLE primarily affects women of childbearing age, with a nine times greater incidence rate in women than in men [7]. As with other studies, there was marked female preponderance [2,4], and our study showed a mean age of 25 years. Patients in our study presented with varied symptoms, mucocutaneous signs being the most prevalent (86.79%) followed by renal (69.81%), and musculoskeletal symptoms (56.60%) which is similar to a study by Kakati et al., 2015 [8]. Zahran et al., 2019 found hematological disease (60%) as the most common clinical manifestation, followed by arthritis (57.1%) and renal disorders (57.1%) [2]. Henriques et al., 2013 also found hematological manifestations as the most common manifestation [9].

All the cases in our study were positive for ANA. In studies done by Zahran et al., 2019 and Kakati et al., 2015, ANA positivity was 54.3% and 96.22% respectively [2,8]. In the present study, out of the 32 cases, 25 cases (78%) were positive for anti-dsDNA antibody, which was similar to that of Zahran et al., 2019 (28 cases, 80%) [2]. However, the positivity in our study was lower as compared to a study from Northeast India by Kakati et al., 2015, who found 96.22% of cases positive for anti-dsDNA antibody [8].

Renal involvement in SLE occurs in 20-50 % of cases during their disease course [10]. In the present study, 43.7% of cases had renal involvement as documented by renal biopsy. Out of these, the majority of the cases (25%) were diagnosed with class IV lupus nephritis as per the International Society of Nephrology/Renal Pathology Society classification. In a study done by Das et al., 2023, the majority of the lupus nephritis cases were Class IV [11]. 

ALC and absolute CD3- cells were decreased in cases as compared to controls in the present study. These findings are consistent with the study of Erkeller-Yüksel et al., 1993 [12].

In SLE patients, the number of CD56(+) NK cells decreases as compared to healthy controls; this is caused by an imbalance in the creation and destruction of different types of immune cells. This seems to be associated with the induction of apoptosis by autoantibodies, primarily related to nuclear-targeting autoantibodies and associated with disease flare [9]. The decline in NK cells, particularly the CD56dim subgroup (mature), could indicate that these extremely toxic cells are migrating from the peripheral circulation to target organs, exacerbating local tissue damage [2].

In the present study, CD56(+) cells were analyzed in both cases and controls. The median was found to be 12.3 for cases and 24.7 for controls. Thus, these data show that the percentage of CD56(+) cells was reduced in cases as compared to controls. A study by Lin et al., 2017 was in agreement with our findings [13]. These findings reiterate the fact that a decrease in CD56+ NK cells in cases of SLE could be attributed to autoantibody-related apoptosis induced by the formation of immune complexes and serum cytokines like IFN-α [9].

Erkeller-Yüksel et al., 1993 found lower levels of CD16(+) NK cells in SLE patients as compared to controls i.e., one-third of control levels (0.1±0.08 vs.0.3±0.1 ×109/l) [12]. They attributed this reduction to immunosuppressive treatment [12]. In the present study, the percentage of CD16+ cells was also observed to be reduced in cases as compared to controls, i.e., two-fifths of the control levels. All the patients were on immunosuppressive therapy. In humans, NK cells constitute approximately 10% of peripheral blood lymphocytes [14]. They can be split into two major subsets, based on the relative densities of CD56 surface expressions [14].

CD56dimNK cells comprise 90% of peripheral blood NK cells, have a high cytolytic capacity, and secrete low levels of cytokines [14]. Conversely, CD56bright NK cells are the main type of NK cell in secondary lymphoid tissues and sites of inflammation, secrete a greater number of cytokines but acquire cytotoxicity only after prolonged activation [14]. CD56dim NK cells express killer immunoglobulin-like receptors (KIR) and CD16, and the CD56bright NK cells lack expression of KIR and CD16 [15]. By upregulating T-cell activation in lymphoid organs and subsequent B-cell responses, CD56brightNK cells may have an impact on autoimmune disorders [15].

We attempted to find cut-off values of ALC, CD3(-) NK cells, CD56(+), and CD16(+) NK cells in SLE patients by ROC curve analysis. For ALC, the area under the ROC curve is 0.924, with a p-value of <0.001, which is considered statistically significant. The optimal cut-off was found to be 2528/cumm for the test with a sensitivity of 96.7% and a specificity of 78.1%. So ALC of < 2528/ cumm can be taken as the cut-off value in SLE.

ROC curve analysis was done for absolute CD3- cells and a cut-off value of 481/cumm with a sensitivity of 86.7% and a specificity of 84.4% was derived for SLE. Thus, in patients of SLE, less than 481/cumm can be taken as the cut-off value. Similarly, a cut-off value of <23% was found statistically significant for CD56(+) NK cells in SLE with 60% sensitivity and 75% specificity. The area under the ROC curve for CD16(+) NK cells was 0.830, with a p-value of <0.001, which is statistically significant. The optimal cut-off was found to be 29% for the test with a sensitivity of 70% and specificity of 87.5%. So, the percentage of CD16(+) cells of < 29% can be taken as the cut-off value in SLE.

A thorough literature search using Google Scholar as the search engine and using keywords such as 'absolute lymphocyte count', 'CD3 negative natural killer cells', 'CD56 natural killer cells', 'CD16 natural killer cells', 'systemic lupus erythematosus', and 'ROC curve' did not reveal any studies giving a cut-off value with sensitivity and specificity. So, to our knowledge, the present study is the first to establish cut-off values of ALC and the different subsets of NK cells in SLE patients.

The literature review reveals a negative correlation of NK cell counts with disease activity i.e., the more severe the disease, the lesser the NK cell count [2,4]. However, we could not ascertain a significant correlation between these cut-off values of NK cells and their subsets with disease activity. This could be explained by the small sample size and the cross-sectional nature of our study. Therefore, a larger study may be helpful to establish the role of NK cells in predicting disease flare using these cut-off values.

In the present study, we classified the cases into no flare, mild/moderate flare, and severe flare according to the SELENA-SLEDAI flare instrument. Out of the 32 cases, 20 presented with mild/moderate flare (62.5%), eight with severe flare (25%), and four with no flare (12.5%). We observed that age, gender, ANA, anti-ds-DNA antibody, ALC, absolute NK cell count, and CD56 and CD16 percentages could not predict disease activity.

In most investigations, SLE patients had a significantly higher number of immature CD56bright NK cells and a significantly lower number of mature CD56dim NK cells as compared to controls. This has been explained by various mechanisms like the release of a greater number of CD56bright NK cells from bone marrow and/ or lymphoid tissue to circumvent high turnover of CD56dim NK cells, CD56bright NK cell resistance to oxidant-induced cell death, and CD56bright NK cell selective expansion [2,15]. However, in the present study, the percentage of immature CD56bright NK cells in SLE patients was less as compared to controls (18.75% vs 40%) and the number of mature CD56dim NK cells was higher in SLE patients as compared to controls (78.13% vs 53.4%). This may be explained by the fact that most of the cases in our study were in mild/moderate flare, where the turnover of CD56dim NK cells may not be increased.

In the present study, there was no significant difference between CD56bright or CD56dim NK cells with age, gender, ANA, anti-ds-DNA antibody, ALC, absolute NK cell count, and CD56 and CD16 percentages or renal involvement. A conclusive interpretation can not be derived from this as the number of cases in each subset of NK cell maturation was too low. Going through the literature, various authors have noted different observations. Henriques et al. discovered that absolute numbers of CD56bright NK and CD56dim NK cells were considerably reduced in patients of SLE with measurable anti-dsDNA antibody levels than in those without anti-dsDNA antibodies [9]. However, we found no substantial difference between the two in our research.

Immature CD56bright NK cells showed positive correlations with anti-dsDNA, according to Zahran et al. [2]. Henriques et al. found no link between CD56bright NK cells and disease activity [9]. Schepis et al. reported no significant difference in CD56bright NK cells between SLE patients in relation to illness severity, anti-double-stranded DNA antibodies, or the presence of nephritis, which is consistent with our findings [15]. Liu et al. found a reduction of the mature CD56dim NK cells and an increase of immature CD56bright NK cells in patients with SLE [16]. However, these findings were not statistically significant. They attributed the low sample size to their findings. They also discovered that the proportions of CD56dim NK cells and CD56bright NK cells were unaffected by illness severity [16]. There is no evidence in the literature with regard to the association of age, gender, ANA, and ALC with the subsets of NK cells in SLE.

Limitations of the study include a smaller sample size. The duration of the study was only one year. So, the follow-up of the patients was limited and the correlation of NK cell count with clinical outcome could not be done.

## Conclusions

NK cells and their subpopulations of CD56(+) and CD16(+) cells are decreased in patients with SLE as compared to controls, signifying that NK cells have a role in shaping the adaptive immune system. ROC curve analysis was determined for NK cells and a cut-off value of <481/cumm with a sensitivity of 86.7% and specificity of 84.4% was obtained.

A statistically significant cut-off point of NK cells that could reflect disease severity could not be established as the number of cases in each category was too low. However, a larger study may be carried out to establish the role of NK cells in predicting disease activity using these cut-off values.
